# Supplementary material for: Molecular analysis of human Ero1 reveals novel regulatory mechanisms for oxidative protein folding
Source: Life Sci Alliance. 2018 Jun 26;1(3):e201800090. doi: 10.26508/lsa.201800090 (PMC6238587; doi:10.26508/lsa.201800090)
Supplement: Supplementary file 1 [file LSA-2018-00090_TableS1.pdf]

Table S1. Electrospray ionization mass spectrometric analysis of purified Ero1α complex. Ero1α co-purifies with PDI and both species, as well as the mixed disulfide species, are observed by mass spectrometry. Ero1α is produced with (phospho)gluconoylation of the N-terminal His tag (Geoghegan et al, 1999), with the gluconoylated species being the major state. For clarity, the other states of the N-terminal His tag (no modification and phosphogluconoylation) are not shown. The data are indicative that 14 of the 15 Cys in Ero1α are in a disulfide state and that a mixed disulfide forms between Ero1α and PDI in the inactive complex.

NEM	Species observed	Expected mass (Da)	Experimental mass (Da)	Δmass	Explanation
-	Gluconoylated Ero1α	53255	53240	-15Da	7 disulfides
	PDI	55425	55423	-2Da	1 disulfide
	Gluconoylated Ero1α-PDI complex	108680	108663	-17Da	8 disulfides
+	Gluconoylated Ero1α	53255	53365	+110Da	One NEM<br>7 disulfides
	PDI	55425	55546	+121Da	One NEM, 2 disulfides
	PDI	55425	55670	+245Da	Two NEM, 2 disulfides
