# Supplementary material for: Molecular analysis of human Ero1 reveals novel regulatory mechanisms for oxidative protein folding
Source: Life Sci Alliance. 2018 Jun 26;1(3):e201800090. doi: 10.26508/lsa.201800090 (PMC6238587; doi:10.26508/lsa.201800090)
Supplement: Supplementary file 2 [file LSA-2018-00090_TableS2.pdf]

Table S2. Electrospray ionization mass spectrometric analysis of purified Ero1β complex. Ero1β co-purifies with PDI and both species, as well as the mixed disulfide species, are observed by mass spectrometry. Ero1β is produced with (phospho)gluconoylation of the N-terminal His tag (Geoghegan et al, 1999), with the gluconoylated species being the major state. For clarity, the other states of the N-terminal His tag (no modification and phosphogluconoylation) are not shown. The data are indicative that 12 of the 14 Cys in Ero1β are in a disulfide state.

NEM	Species observed	Expected mass (Da)	Experimental mass (Da)	Δmass	Explanation
-	Gluconoylated Ero1β	51457	51444	-13Da	6 disulfides
	PDI	55425	55421	-4Da	2 disulfides
	Gluconoylated Ero1β-PDI complex	106882	106867	-15Da	7 disulfides
+	Gluconoylated Ero1β	51457	51569	+112Da	One NEM 6 disulfides
	Gluconoylated Ero1β	51457	51694	+237Da	Two NEM 6 disulfides
	PDI	55425	55546	+121Da	One NEM, 2 disulfides
	PDI	55425	55670	+245Da	Two NEM, 2 disulfides
