# Supplementary material for: Molecular analysis of human Ero1 reveals novel regulatory mechanisms for oxidative protein folding
Source: Life Sci Alliance. 2018 Jun 26;1(3):e201800090. doi: 10.26508/lsa.201800090 (PMC6238587; doi:10.26508/lsa.201800090)
Supplement: Supplementary file 3 [file LSA-2018-00090_TableS3.pdf]

Table S3. Plasmids used in this study.

Vector	Background	Promoter	Expressed proteins
pKMK95[a]	pMAL-c2x	tac	H_6-Ero1α (E24-H468)
pAM87[b]	pMAL-c2x	tac	H_6-Ero1α
pKMK209	pMAL-c2x	tac	H_6-Ero1α, ERp57
pAM119	pMAL-c2x	tac	H_6-Ero1α C166A
pKMK123	pMAL-c2x	tac	H_6-Ero1α C94/99A
pKMK126	pMAL-c2x	tac	H_6-Ero1α C99/104A
pKMK128	pMAL-c2x	tac	H_6-Ero1α C104/131A
pKMK130	pMAL-c2x	tac	H_6-Ero1α C94/99/104/131A
pAM230	pMAL-c2x	tac	H_6-Ero1α C208/241A
pAM2[c]	pMAL-c2x	tac	H_6-Ero1β (Q34-R467, natural D129V/H465Q variant)
pAM215[d]	pMAL-c2x	tac	H_6-Ero1β
pAM216	pMAL-c2x	tac	H_6-Ero1β, ERp57
pAM6	pMAL-c2x	tac	H_6-Ero1β C165A
pAM218	pMAL-c2x	tac	H_6-Ero1β C90/95A
pAM219	pMAL-c2x	tac	H_6-Ero1β C95/100A
pKMK212	pMAL-c2x	tac	H_6-Ero1β C100/130A
pAM220	pMAL-c2x	tac	H_6-Ero1β C90/95/100/130A
pOLR130	pET23	T7	H_6-PDI (D18-L508)
pAP1	pET23	T7	H_6-PDIp (Q22-L525)
pKMK177	pET23	tac	H_6-ERp57 (S25-L505)
pHIA282	pET23	T7	H_6-ERp46 (R33-L432, natural variant R354K)
pHIA64	pET23	T7	H_6-ERp72 (V21-L645)
pAM194	pET23	tac	H_6-P5 (L20-L440)
pAM139	pET23	tac	H_6T-PDI (D18-L508)
pAM172	pET23	tac	H_6T-PDI3FLAG
pAM227	pET23	tac	H_6-tDsbC-PDIp (Q22-L525)
pAM225	pET23	tac	H_6-tDsbC-ERp57 (S25-L505)
pAM226	pET23	tac	H_6-tDsbC-ERp46 (R33-L432, natural variant R354K)
pAM223	pET23	tac	H_6-tDsbC-ERp72 (V21-L645)
pAM224	pET23	tac	H_6-tDsbC-P5 (L20-L440)
pMJS333	pET23	tac	H_6-Erv1p (M1-H189)
pMJS205	pLysS	tac	Erv1p, PDI (D18-L508)
pAM104	pLysS	tac	Erv1p, PDI (D18-L508) C56A
pKMK221	pLysS	tac	Erv1p, PDI (D18-L508) C400A
pAM110	pLysS	tac	Erv1p, PDI (D18-L508) C53M/56A/397M/400A

[a]All Ero1α variants in the list are based on this vector; that is, they span the same residues.

[b]XhoI site restored to MCS to pKMK95 to prepare polycistronic vectors.

[c]All Ero1β variants in the list are based on this vector; that is, they span the same residues and contain the same natural mutation.

[d]XhoI site restored to MCS to pAM2 to prepare polycistronic vectors.

Abbreviations: H_6, His tag; H_6T, His tag followed by a TEV protease cleavage site; H_6-tDsbC, His-tagged truncated DsbC followed by a TEV protease cleavage site.
