# Supplementary material for: Molecular analysis of human Ero1 reveals novel regulatory mechanisms for oxidative protein folding
Source: Life Sci Alliance. 2018 Jun 26;1(3):e201800090. doi: 10.26508/lsa.201800090 (PMC6238587; doi:10.26508/lsa.201800090)
Supplement: Supplementary file 4 [file LSA-2018-00090_TableS4.pdf]

Table S4. Biophysical attributes of purified proteins from the ProtParam tool (Gasteiger et al, 2005; https://web.expasy.org/protparam/).

Protein produced	Molecular weight (Da)	Extinction coefficient (M^{-1} cm^{-1})
H_6-Ero1α-PDI	108502.3	115960
H_6-Ero1α C104/131A-PDI	108438.1	115835
H_6-Ero1α C166A	53045.0	70205
H_6-Ero1α C208/241A	53012.9	70080
H_6-Ero1β-PDI	106703.7	117450
H_6-Ero1β C100/130A-PDI	106639.6	117325
H_6-PDI (D18-L508)	56379.2	45755
H_6-PDIp (Q22-L525)	56919.4	44140
H_6-ERp57 (S25-L505)	55350.4	45185
H_6-ERp46 (R33-L432, natural variant R354K)	45545.1	69620
H_6-ERp72 (V21-L645)	71756.7	68105
H_6-P5 (L20-L440)	46170.9	71765
H_6-Erv1p (M1-H189)	22593.5	42315
PDI (D18-L508)[a]	55649.4	45755
PDI3FLAG[a]	58634.3	50225
PDIp (Q22-L525)[a]	56199.7	44140
ERp57 (S25-L505)[a]	54620.6	45185
ERp46 (R33-L432, natural variant R354K)[a]	44815.4	69620
ERp72 (V21-L645)[a]	71027.0	68105
P5 (L20-L440)[a]	46526.3	71765

[a]These constructs contain additional amino acids SHM in the N-terminus resulting from TEV protease cleavage.
